# Determinants on an efficient cellulase recycling process for the production of bioethanol from recycled paper sludge under high solid loadings

**DOI:** 10.1186/s13068-018-1103-2

**Published:** 2018-04-16

**Authors:** Daniel Gomes, Miguel Gama, Lucília Domingues

**Affiliations:** Centre of Biological Engineering, University of Minho, Campus de Gualtar, 4710-057 Braga, Portugal

**Keywords:** Recycled paper sludge, Cellulase recycling, Process intensification, Enzyme thermostability, Enzyme activity phase distribution, Cellulosic bioethanol

## Abstract

**Background:**

In spite of the continuous efforts and investments in the last decades, lignocellulosic ethanol is still not economically competitive with fossil fuels. Optimization is still required in different parts of the process. Namely, the cost effective usage of enzymes has been pursued by different strategies, one of them being recycling.

**Results:**

Cellulase recycling was analyzed on recycled paper sludge (RPS) conversion into bioethanol under intensified conditions. Different cocktails were studied regarding thermostability, hydrolysis efficiency, distribution in the multiphasic system and recovery from solid. Celluclast showed inferior stability at higher temperatures (45–55 °C), nevertheless its performance at moderate temperatures (40 °C) was slightly superior to other cocktails (ACCELLERASE^®^1500 and Cellic^®^CTec2). Celluclast distribution in the solid–liquid medium was also more favorable, enabling to recover 88% of final activity at the end of the process. A central composite design studied the influence of solid concentration and enzyme dosage on RPS conversion by Celluclast. Solids concentration showed a significant positive effect on glucose production, no major limitations being found from utilizing high amounts of solids under the studied conditions. Increasing enzyme loading from 20 to 30 FPU/g_cellulose_ had no significant effect on sugars production, suggesting that 22% solids and 20 FPU/g_cellulose_ are the best operational conditions towards an intensified process. Applying these, a system of multiple rounds of hydrolysis with enzyme recycling was implemented, allowing to maintain the steady levels of enzyme activity with only 50% of enzyme on each recycling stage. Additionally, interesting levels of solid conversion (70–81%) were also achieved, leading to considerable improvements on glucose and ethanol production comparatively with the reports available so far (3.4- and 3.8-fold, respectively).

**Conclusions:**

Enzyme recycling viability depends on enzyme distribution between the solid and liquid phases at the end of hydrolysis, as well as enzymes thermostability. Both are critical features to be observed for a judicious choice of enzyme cocktail. This work demonstrates that enzyme recycling in intensified biomass degradation can be achieved through simple means. The process is possibly much more effective at larger scale, hence novel enzyme formulations favoring this possibility should be developed for industrial usage.

## Background

Over the last decades, lignocellulosic ethanol assumed a major role on the definitive affirmation of biofuels in the new global energy picture. Relying on cheaper raw materials, such as agro-forestry wastes, it can represent an important boost for the economy of small and local communities [1]. Additionally, it may also encompass the utilization of industrial/municipal wastes, enabling some value recovery from a negative-cost material and a reduction on its environmental impact.

Despite the notorious progresses made, the development of suitable hydrolytic enzymes still faces challenges, such as the high cost and sensitivity to process conditions.

Distinct estimations for the cost of cellulases have been pointed out by different studies. According to Klein-Marcusschamer et al. [2], the cellulase cost on ethanol production is approximately $ 0.68 per gallon, close to $ 0.5 per gallon suggested by Novozymes [3]. Aden and Foust [4], however, already reported a value around $ 0.1 per gallon, similar to $ 0.3 reported by Lynd et al. [5] and $ 0.32 reported by Dutta et al. [6]. Even though important reductions have been achieved on their production cost, operated by intense research from both industry and academia, some authors already admitted these strategies will not allow much further reductions. Independently of the current cost of enzymes, it is widely recognized as a critical determinant for cellulosic ethanol competitiveness.

A reduction on cellulase cost has been intensively pursued through different strategies, being one of them the reutilization of enzymes [7]. This has been achieved by distinct ways: recovering enzymes by ultrafiltration [8–11]; re-adsorption of free enzymes into fresh solid [12–16]; finally, partial recycling of whole final medium, and consequently, of the enzymes [17]. While less complex, the two later options present limitations that can severely hamper an efficient recovery process. Re-adsorption into fresh solid requires that a significant fraction will efficiently adsorb over the process of solid separation. Also, low cellulose-binding enzymes, such as β-glucosidase, would require being supplemented [17–19]. On the other hand, partial/total whole medium (solid and liquid) recycling will always be restricted by lignin build-up constraints and the consequent increase of non-productive enzyme binding [20]. As an alternative, ultrafiltration can allow an efficient separation of enzymes that can then be directly applied on a new hydrolysis process. In addition to being potentially more expensive, the late approach requires the enzymes to be freely available in the liquid phase, i.e., they should have low affinity towards the final solid residue. Hence, a critical role is attributed to the composition and structure of the raw material but also to the selected cellulases. Both have shown to significantly affect the specific distribution of free (soluble) and solid-bound (adsorbed) enzymes as well as the effectiveness of their recovery [7, 9]. Enzymes adsorbed to the solid can still be recovered by pH switch [14, 21, 22] or using different chemicals [15, 23]. Therefore, it seems clear that the binomial substrate–enzyme will determine the most suitable recycling strategy for each case.

In the scope of a more economic process, also intensification has been pursued from multiple angles, namely through an increase on solid loadings [24, 25] or through an optimized integration of hydrolysis and fermentation [26–28]. For high-water retention materials, such RPS (recycled paper sludge), converting high solid loadings represents, however, a serious challenge as enzymes have a reduced mobility due to a lower free liquid in suspension. In fact, Marques et al. [29] reported 17.9% RPS as the maximum solid concentration that enabled hydrolysis. Considering the moderate levels of cellulose and hemicellulose in this material, maximizing sugar concentration on the final hydrolysate is critical for a sustainable process. On the other hand, this should also be taken into account when selecting and designing a cellulase recycling strategy. High solid loadings and/or materials with a high lignin content could be a serious challenge, particularly when solid is recycled.

Here we perform a structured and sequential study on the implementation of cellulase recycling in the process of bioethanol production from recycled paper sludge under high solid loadings. The performance of different cellulase cocktails is addressed in terms of hydrolytic performance, stability and final enzyme recovery. Aiming at process intensification, the effect of higher amounts of solid and enzyme on the hydrolysis efficiency is studied, to find the best operational conditions. Those were then considered on the implementation of a system of multiple rounds with cellulase recycling where the levels of enzyme activity and solid conversion were evaluated.

## Methods

### Enzymes, substrate and microorganisms

Enzymatic hydrolysis assays were conducted separately with different cellulase cocktails: Celluclast 1.5 L (from Novozymes A/S); ACCELLERASE^®^ 1500^1^^,^ 2 (from DuPont); Cellic^®^ CTec2 (from Novozymes A/S). FPase activity of these preparations was determined to be 60, 40 and 120 FPU/mL, respectively. Also, pNPG β-glucosidase activities were determined as 42, 499 and 3609 U/g, respectively. The protein content assessed by Bradford assay (using BSA as standard) were 30, 20 and 58 mg/g, respectively.

Due to the low level of β-glucosidase activity found on Celluclast, this cocktail was always supplemented with the β-glucosidase preparation Novozyme 188 (from Novozymes A/S) on a β-glucosidase/FPase ratio of 3.

Recycled paper sludge (RPS) was kindly provided by RENOVA (Torres Novas, Portugal) and refers to the residue obtained from the wastewater treatment of paper recycling effluents generated by this company. Due to its high carbonate content, which results on an alkaline solid with a reduced holocellulose fraction, prior to its utilization RPS was treated with hydrochloric acid 37% and then washed, first with water and then with buffer (0.1 M acetic acid/sodium acetate) [8]. This resulted on a neutralized RPS (nRPS), which was used in the current work, with an increased holocellulose fraction: 27.1% cellulose, 7.3% xylan and 65.7% acid-insoluble solid.

Fermentations were conducted with *Saccharomyces cerevisiae* CA11, a strain which was recently reported to have a good fermentation performance at high temperatures [30, 31].

### Thermostability assays

To assess which cellulase mixture is more stable towards thermal deactivation, the efficiency of nRPS (carbonates-neutralized RPS) solid conversion was quantified after enzymes exposure to increasing periods of incubation at different temperatures (45, 50 and 55 °C). Then, after the pre-incubation period, nRPS hydrolysis for 18 h, with 5% (w/v) solids at 50 °C, was performed to evaluate the remaining activity.

### Comparative hydrolysis efficiency and enzyme activity phase distribution of different cellulase mixtures

To enable a direct comparison of the performance of the three cellulase mixtures, their profiles of glucose production were studied using two distinct solid concentrations [10 and 18% (w/v)]. For that purpose, the solid suspension was incubated with a volume of enzyme equivalent to 20 FPU/g cellulose in 0.1 M sodium acetate/acetic acid buffer (pH of 4.8) and incubated at 40 °C for 96 h.

To evaluate activity distribution of the three cellulase mixtures in the multiphasic system, Cel7A (major cellulase component of *Trichoderma reesei* cocktails) levels were quantified in both the solid and liquid fractions, both after hydrolysis and alkaline washing [21].

### Effect of solid concentration and enzyme loading on the efficiency of nRPS hydrolysis

The effect of both solid concentration and enzyme loading on the efficiency of nRPS hydrolysis was studied conducting a central composite inscribed (CCI) design. Each factor was tested for five levels for the nominal values of − 1, − 0.7, 0, + 0.7 and + 1. Solid concentration was tested in the range of 14–22% (w/v), defined according to preliminary tests on the mixing efficiency as a function of nRPS consistency. Enzyme loading was set to the range of 20–30 FPU/g cellulose. The lower level is within the usual values employed on the literature [7, 8, 32, 33]. The upper level is slightly superior to evaluate potential improvements on enzyme hydrolysis efficiency. In the context of enzyme recycling, the overall enzyme load is actually reduced, as only a fraction of the initial load is used in the subsequent cycles.

The matrix of the CCI design with both the nominal and the real values is presented in Table 1.

**Table 1:** CCI design matrix presenting the normalized and the real values for each run

Run	Normalized value		Real value	
	*X*_1_	*X*_2_	*X*_1_ [% (w/v)]	*X*_2_ (FPU/g_cellulose_)
1	− 1	− 1	14	20
2	− 1	0	14	25
3	− 1	+ 1	14	30
4	0	+ 1	18	30
5	+ 1	+ 1	22	30
6	+ 1	0	22	25
7	+ 1	− 1	22	20
8	0	− 1	18	20
9	0	0	18	25
10	0	0	18	25
11	0	0	18	25
12	0	0	18	25
13	− 0.7	− 0.7	15.2	21.5
14	− 0.7	+ 0.7	15.2	28.5
15	+ 0.7	+ 0.7	20.8	28.5
16	+ 0.7	− 0.7	20.8	21.5

*X*_*1*_ nRPS solid concentration, *X*_*2*_ enzyme dosage

### Multiple rounds of hydrolysis with enzyme recycling

Enzymatic hydrolysis in the context of cellulase recycling was conducted similar to the single-round experiments. For the first round, the sterilized solid suspension [22% (w/v)] was mixed with 20 FPU/g cellulose of Celluclast (complemented with *β*-glucosidase) and incubated for 120 h (40 °C; 200 rpm). Afterwards, this mixture was inoculated with 8 g/L (fresh biomass) CA11 yeast cells and incubated for 24 h at 35 °C.

At the end of the round, final broth was centrifuged (9000 rpm for 20 min) to separate fractions. Supernatant, containing free enzymes (in the liquid fraction), was filtered through a 0.22-μm polyethersulfone (PES) filter to remove impurities and stored (4 °C) until further use. The solid was subjected to an alkaline washing, as previously described by Gomes et al. [8]. The elution liquid, containing the desorbed enzymes, was filtered to remove major impurities and stored until use. Prior to its storage, the pH of this liquid was adjusted to the common operational pH (4.8) through the addition of 1 M acetic acid/sodium acetate buffer (pH 4.8). Final solid was repeatedly washed, oven dried (at 45 °C) until an estimated water content below 10% was reached, and finally stored until final analysis.

**Fig. 1:**
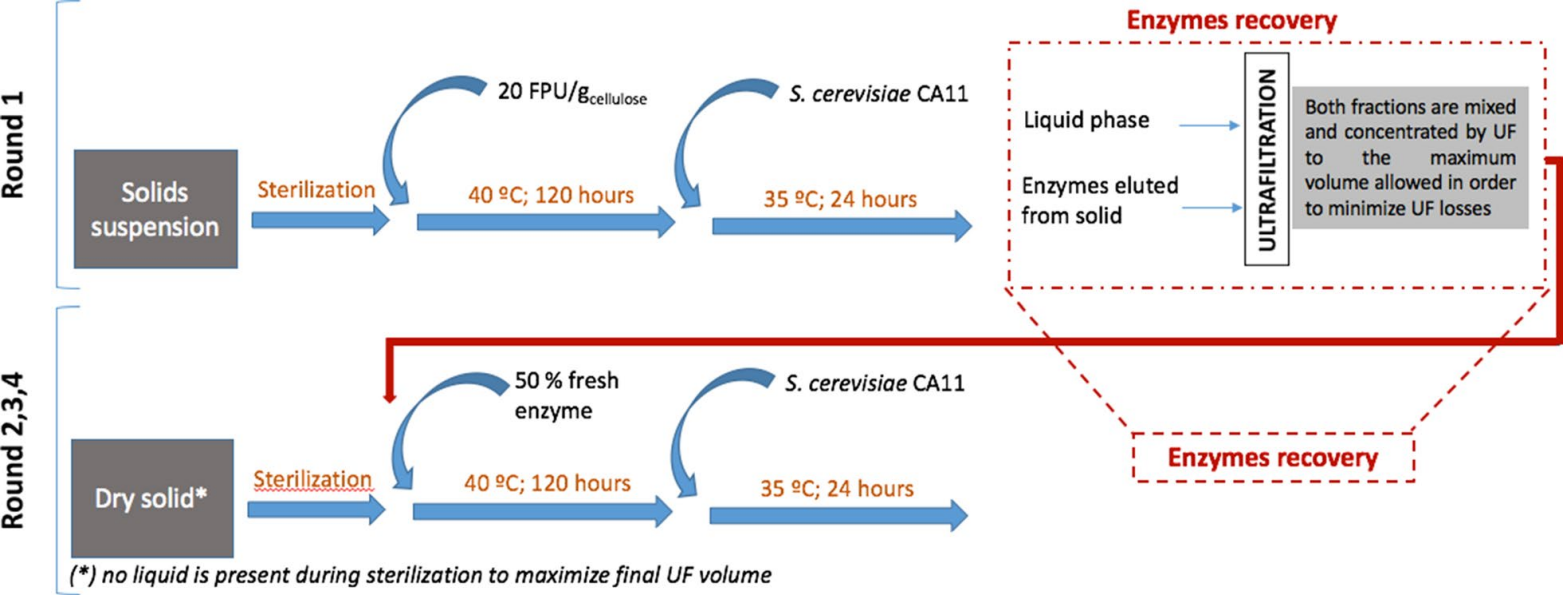
Schematic representation for the system of multiple rounds of hydrolysis (and fermentation) with cellulase recycling

For cellulase recycling, both enzyme-containing fractions (stored at 4 °C) were mixed and concentrated using a tangential ultrafiltration system Pellicon XL membrane with a 10 kDa cut-off PES membrane (Millipore, Billerica, MA, USA). The two fractions were initially concentrated by diafiltration, and at the end, adjusted to a final fixed volume. For a new round of hydrolysis, the freshly sterilized solid was resuspended on the enzyme suspension obtained from the previous ultrafiltration procedure, filter-sterilized with 0.2-μm PES syringe filters. For each recycling stage, a portion of fresh enzyme was added to this suspension, corresponding to 50% of the original enzyme dosage (maintaining the β-glucosidase/FPase activity ratio). The new solid suspension was then subjected to the same conditions of hydrolysis and fermentation, as previously described. This procedure was applied over a total of four rounds of hydrolysis and fermentation as schematically described on Fig. 1.

### Analytical procedures

#### Sugars and ethanol quantification

After thawing, aliquots from hydrolysis and fermentation experiments were diluted, filtered and then analyzed by high-performance liquid chromatography (HPLC) for glucose and ethanol quantification. Samples were eluted on a Varian MetaCarb 87H column at 60 °C, with 0.005 M H_2_SO_4_ at a flow rate of 0.7 mL/min, and a refractive index detector.

#### Measurement of enzymatic activity

Samples collected for quantification of enzymatic activity were stored at 4 °C until further utilization. Cel7A, Cel7B and β-glucosidase activities were quantified by fluorescence spectroscopy with slight differences according to the specific cellulolytic component, following a modified version of the protocol previously published by Bailey and Tähtiharju [34]. For Cel7A, Cel7B and β-glucosidase quantification, 400 μL of a freshly prepared solution of 1 mM 4-methylumbelliferyl-b-d-cellobioside (MUC, Sigma-Aldrich, M6018), 4-methylumbelliferyl-b-d-lactopyranoside (MULac, Sigma-Aldrich, M2405) and 4-methylumbelliferyl-b-d-glucopyranoside (MUGlc, Sigma-Aldrich, M3633), respectively, were mixed with 50 μL of enzyme sample (properly diluted on buffer considering the linearity range of the method) and then incubated for 15 min at 50 °C. After that, the reaction was stopped by the addition of 550 μL of 1 M Na_2_CO_3_ and measured on a black bottom 96-well UV fluorescence microplate using a Biotech Synergy HT Elisa plate reader. For Cel7B quantification, the addition of 50 μL of a mixture containing 1 M glucose and 50 mM cellobiose is still required to inhibit Cel7A and β-glucosidase activities. Cel7A, Cel7B and β-glucosidase act on their specific substrates releasing free 4-methylumbelliferone (MU, Sigma-Aldrich, M1508), which results on a change of the fluorescence spectra that is quantified for an excitation and emission wavelengths of 360 and 460 nm, respectively.

#### Determination of solid composition

The solid main composition, either corresponding to the initial material or after enzymatic hydrolysis, was determined by quantitative acid hydrolysis [35]. After oven drying (at 45 °C) to a water content inferior to 10%, approximately 0.5 g of solid was mixed with 5 mL of 72% (w/v) H_2_SO_4_ for 1 h at 30 °C. Afterwards, this mixture was subjected to a dilute hydrolysis by raising the volume with water to a total mass of 148.67 g and subsequently autoclaved for 1 h at 121 °C. Next, the solid residue was recovered by filtration (cresol Gooch no. 3) and dried (at 105 °C) until constant weight. Different sugar monomers formed during hydrolysis were quantified by HPLC analysis of the liquid fraction.

#### Estimation of hydrolysis and fermentation yields

For an overall assessment of hydrolysis and fermentation processes, glucose and ethanol production yields (GY_120_ and EY_23_, respectively) were estimated according to the following equations:

$${\text{G}}{{\text{Y}}_{120}}\left( \% \right) = \frac{{{{\left[ {\text{Glucose}} \right]}_{120}} + 1.053{{\left[ {\text{Cellobiose}} \right]}_{120}}}}{{1.111{{\left[ {\text{Solids}} \right]}_{\text{i}}} \times {F_{\text{cel}}}}} \times 100$$

$${\text{E}}{{\text{Y}}_{23}}\left( \% \right) = \frac{{{{\left[ {\text{Ethanol}} \right]}_{23}}}}{{0.51\left( {1.111{{\left[ {\text{Solids}} \right]}_{\text{i}}} \times {F_{\text{cel}}} \times 0.963} \right)}} \times 100$$

where [Glucose]_120_ and [Cellobiose]_120_ are the concentrations of glucose and cellobiose, respectively, at 120 of hydrolysis and [Ethanol]_23_ is the ethanol concentration at 23 h of fermentation. [Solids]_i_ refers to the initial concentration of dry solid and *F*_cel_ is the fraction of cellulose on a dry solid base. 1.111 consists on the glucan to glucose conversion ratio, 0.51 is the maximum theoretical conversion of glucose into ethanol and 0.963 was the dilution factor imposed by cells inoculation.

## Results and discussion

On a recent work it was demonstrated that nRPS can be used for bioethanol production, and additionally, is suitable for the implementation of a cellulase recycling system [8]. As a proof-of-concept approach, these tests were, however, conducted under non-intensified conditions [5% (w/v) solids; hydrolysis temperature of 35 °C].

Here we have addressed two important factors targeting the scalability and the economic feasibility of the process, either in terms of nRPS solid conversion but also on the integration of an enzyme recycling system: the selection of the cellulase cocktail and the intensification of solid conversion.

### Thermostability of different cellulase mixtures

**Fig. 2:**
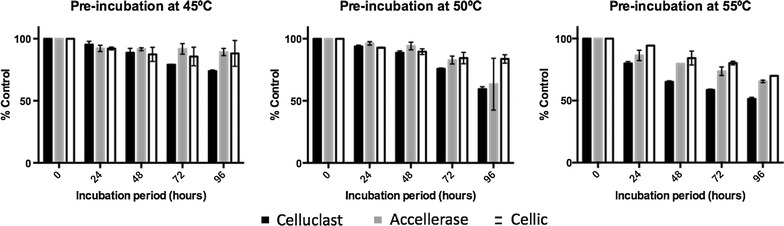
Variation of solid conversion by different cellulase mixtures after increasing periods of pre-incubation at different temperatures

Considering that optimal enzymatic hydrolysis occurs around 50 °C, increased thermostabilities represent an important feature in the context of enzyme reutilization. Figure 2 presents the variation of nRPS solid conversion after incubation of the cellulase suspension at 45, 50 and 55 °C, for different time periods.

As expected, all cocktails presented an increasing loss of hydrolysis capacity with cumulative periods of incubation, being this behavior more prominent for higher temperatures. As an example, for an incubation at 45 °C, after 72 h of incubation the conversion degree still remained above 78% for all cocktails comparatively to the control levels. On the other hand, for a temperature of 55 °C, the conversion dropped to 59, 74 and 80% for Celluclast 1.5 L (Celluclast), ACCELLERASE^®^ 1500 (Accellerase) and Cellic^®^ CTec2 (Cellic), respectively. Differences on thermal deactivation between cocktails were minor for the smallest periods of incubation, excepting for the study at 55 °C, where some differences are already found on an early stage. Considering an incubation period equal or higher to 48 h, significant differences are visible. The hydrolysis efficiency of Celluclast was significantly more affected comparing to Accellerase or Cellic. It is worth noting, however, that the absolute values of glucose production were 4–21% higher for the case of Celluclast, as described in more detail in the next section.

### Hydrolysis efficiency of different cellulase cocktails

**Fig. 3:**
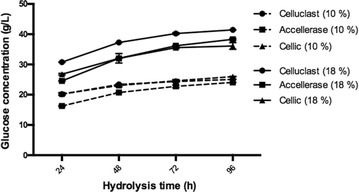
Profiles of glucose production using distinct enzyme mixtures under different solid concentrations, at 40 °C

Thermal deactivation assays were not enough to clearly identify the most suitable cellulase cocktail to be employed at moderate-high temperatures. Although Celluclast present an inferior resistance to thermal denaturation, it enabled higher values of solid conversion. Therefore, and considering the notorious reduction of activity observed in the range of 45–55 °C, which may be especially critical on a cellulase recycling context, the profiles of glucose production obtained by the three cocktails were evaluated for a temperature of 40 °C at different solid concentrations (Fig. 3). Thermal denaturation tests conducted with Celluclast at 40 °C on a week-long experiment provided indications of no activity loss under these conditions.

For a solid concentration of 10% there was not a significant difference on solid conversion between cocktails although Accellerase presented a slightly inferior performance on the first 48–72 h. On the other hand, for 18% solids, Celluclast enabled an average 15% higher glucose production over the entire hydrolysis period, comparatively to the other cocktails. These results suggest that at moderate temperatures (40 °C) where thermal denaturation is low or absent, both Accellerase and Cellic could not surpass Celluclast. It is worth to mention that even supplemented with Novozyme 188, β-glucosidase levels on Celluclast assays are considerably inferior comparatively to the other cocktails: 4.11 U/mL for Celluclast; 13.53 and 37.41 U/mL for Accellerase and Cellic, respectively. This seems to confirm that on this set of conditions (enzyme and solid loadings) the levels of β-glucosidase are not limiting the hydrolysis, as suggested by the absence of cellobiose accumulation (data not shown), hence it does not represent a relevant factor for the different performances.

On these particular conditions, Celluclast seemed to present a slight advantage over the other cocktails regarding hydrolysis performance, nevertheless, enzyme distribution between phases still needed to be assessed.

### Phase activity distribution and efficiency of alkaline washing

The final activity distribution among solid and liquid fractions is critical for enzyme recycling and process complexity. Even though lignin represents nearly 20% of RPS composition [29], being commonly reported as an efficient enzyme adsorbent (by non-productive binding), it was recently observed that 70% of final Cel7A activity is found on the liquid fraction after hydrolysis of RPS with Celluclast under 5% (w/v) solids [8]. This represents a good scenario for enzyme reutilization, as a significant part of the activity is easily recovered.

As reported by other authors [7, 33], different cellulase mixtures may display diverse solid–liquid distributions. To enable the evaluation of the different cellulase mixtures behavior in this regard, Cel7A levels were quantified on both liquid and solid fractions after hydrolysis and alkaline washing, used to extract the adsorbed enzyme (Table 2).

**Table 2:** Final distribution of Cel7A activity after hydrolysis of nRPS and alkaline washing using different cellulase mixtures

Initial activity (IU/mL)	Celluclast		Accellerase		Cellic	
	7.837 ± 0.341		18.107 ± 0.102		15.003 ± 0.411	
	Activity level (IU/mL)	Fraction (%)^b^	Activity level (IU/mL)	Fraction (%)^b^	Activity level (IU/mL)	Fraction (%)^b^
Activity after hydrolysis						
Liquid	4.566 ± 0.508	61.3	12.646 ± 0.361	62.9	6.031 ± 0.100	40.1
Solid	2.077 ± 0.121	38.7	5.391 ± 0.056	37.1	6.519 ± 0.596	59.9
Alkaline washing						
Liquid	1.381 ± 0.077	60.2	4.281 ± 0.038	52.5	4.194 ± 0.081	41.2
Solid	0.651 ± 0.071	39.8	2.791 ± 0.020	47.5	4.331 ± 0.125	58.8
Overall recovery (%)^a^	87.9		80.8		60.2	

Hydrolysis was conducted for 96 h with 18% solids and 20 FPU/g_cellulose_ at 40 °C

^a^Refers to the sum of the free enzymes on the liquid phase after hydrolysis and alkaline elution

^b^Refers to the fraction of the total number of IUs found on each fraction

First, it is worth noting that significant differences were observed regarding the initial levels of Cel7A for the different cocktails even though the same FPU activity was applied on every case. This suggests differences on the composition of each cocktail and on its synergetic mechanisms of enzymatic hydrolysis. Taking into account the values of Cel7A activity one can observe that Celluclast and Accellerase distribute similarly among fractions, with 61.3 and 62.9% of total final activity being found on the liquid fraction, respectively. A significant part still remains adsorbed to the final solid, hampering a more efficient enzyme reutilization. In what concerns Cellic mixture, the enzyme levels on solid fraction were even higher, close to 60% of the final activity. Similarly, different efficiencies were also attained for alkaline washing: 60, 53 and 41% of the enzymes were recovered for Celluclast, Accellerase and Cellic, respectively. As the performances of the different cocktails did not vary considerably (and consequently the final solid composition), no major differences on enzyme fractionation are expected due specifically to distinct binding affinities to cellulose and lignin [9]. On the other hand, these results seem to suggest that different cellulase preparations can, in fact, present very distinct enzyme fractionation profiles for the same material, possibly due to different binding affinities associated to enzymes from different sources. A similar difference was observed by Rodrigues et al. [33] for Celluclast and Cellic binding during the hydrolysis of wheat straw: 26–28% of original Cel7A activity was found soluble on the final liquid fraction on Celluclast; final soluble Cel7A for Cellic was only around 6%. Also, a recent study conducted by Strobel et al. [36] have demonstrated that specific mutations on the *T. reesei* Cel7A CBM can cause significant differences on the binding affinity to both cellulose and lignin, confirming the determinant role of enzyme properties on its binding mechanism to distinct fractions of the solid.

As it can be seen from Table 2, it was possible to achieve an overall recovery of final activity in the range of 60%, for Cellic, 81% for Accellerase and 88%, for Celluclast. Thus, the two later cocktails may be recycled to larger extent, potentially enabling important savings.

### Effect of nRPS concentration and cellulase loading

Even though nRPS is a residue currently with a negative price associated to disposal costs, maximization of solid concentration should still be pursued, as more concentrated hydrolysates allow higher productivities and lower process costs (e.g., distillation). Preliminary studies indicated that a maximum level of 22% (w/v) in solid consistency can be used, still enabling the “liquefaction” of fibers through enzymes action. For higher amounts of solid, a very high viscosity suspension is obtained which enzymes are unable to process.

Considering the results from previous sections—thermostability, hydrolysis efficiency and distribution in the heterogeneous system (recyclability)—Celluclast was chosen for a CCI design studying the influence of enzyme loading and solid concentration on the nRPS hydrolysis (Table 3).

**Table 3:** Experimental values obtained from a CCI design testing different levels of solid concentration and enzyme loadings

Run	g_solids_/mL_liquid_ (%)	FPU/g_cellulose_	Glu_120_ (g/L)	Eth_23_ (g/L)	GY_120_ (%)	EY_23_ (%)
1	14.0	20.0	34.8	15.7	84.7	78.0
2	14.0	25.0	36.3	17.4	88.4	86.2
3	14.0	30.0	38.0	18.8	92.6	93.1
4	18.0	30.0	46.7	24.1	88.4	92.9
5	22.0	30.0	58.9	29.4	91.3	92.8
6	22.0	25.0	56.6	28.7	87.6	90.7
7	22.0	20.0	52.4	26.8	81.2	84.5
8	18.0	20.0	44.0	21.2	83.3	81.6
9	18.0	25.0	47.1	21.5	89.1	83.0
10	18.0	25.0	44.8	22.1	84.8	85.2
11	18.0	25.0	46.3	22.4	87.7	86.3
12	18.0	25.0	46.3	22.3	87.7	85.9
13	15.2	21.5	38.3	18.6	85.8	85.0
14	15.2	28.5	40.6	19.9	91.0	90.9
15	20.8	28.5	52.6	26.9	86.2	89.6
16	20.8	21.5	51.1	24.6	83.7	82.2

From the results of the CCI design, four distinct variables of response were fitted to the experimental data through a second-order polynomial model: glucose concentration (Glu_120_) and production yield (GY_120_) after 120 of hydrolysis; ethanol concentration (Eth_23_) and production yield (EY_23_) after 23 h of fermentation (Eth_23_). The models representing the variables of response as a function of the normalized values of solid concentration (*X*_1_) and enzyme loading (*X*_2_) are presented on the Eqs. 1–4.

1 $${\text{Gl}}{{\text{u}}_{120}} = 45.955 + 9.560{X_1} + 1.891{X_2} + 0.515{X_1}^2 - 0.584{X_2}^2 + 0.573{X_1}{X_2}$$

2 $${\text{G}}{{\text{Y}}_{120}} = 87.025 - 1.322{X_1} + 3.557{X_2} + 1.153{X_1}^2 - 1.017{X_2}^2 + 0.165{X_1}{X_2}$$

3 $${\text{Et}}{{\text{h}}_{23}} = 22.212 + 5.285{X_1} + 1.391{X_2} + 0.512{X_1}^2 - 0.087{X_2}^2 + 0.012{X_1}{X_2}$$

4 $${\text{E}}{{\text{Y}}_{23}} = 87.762 + 0.975{X_1} + 5.533{X_2} + 1.551{X_1}^2 + 0.414{X_2}^2 - 1.207{X_1}{X_2}$$

**Fig. 4:**
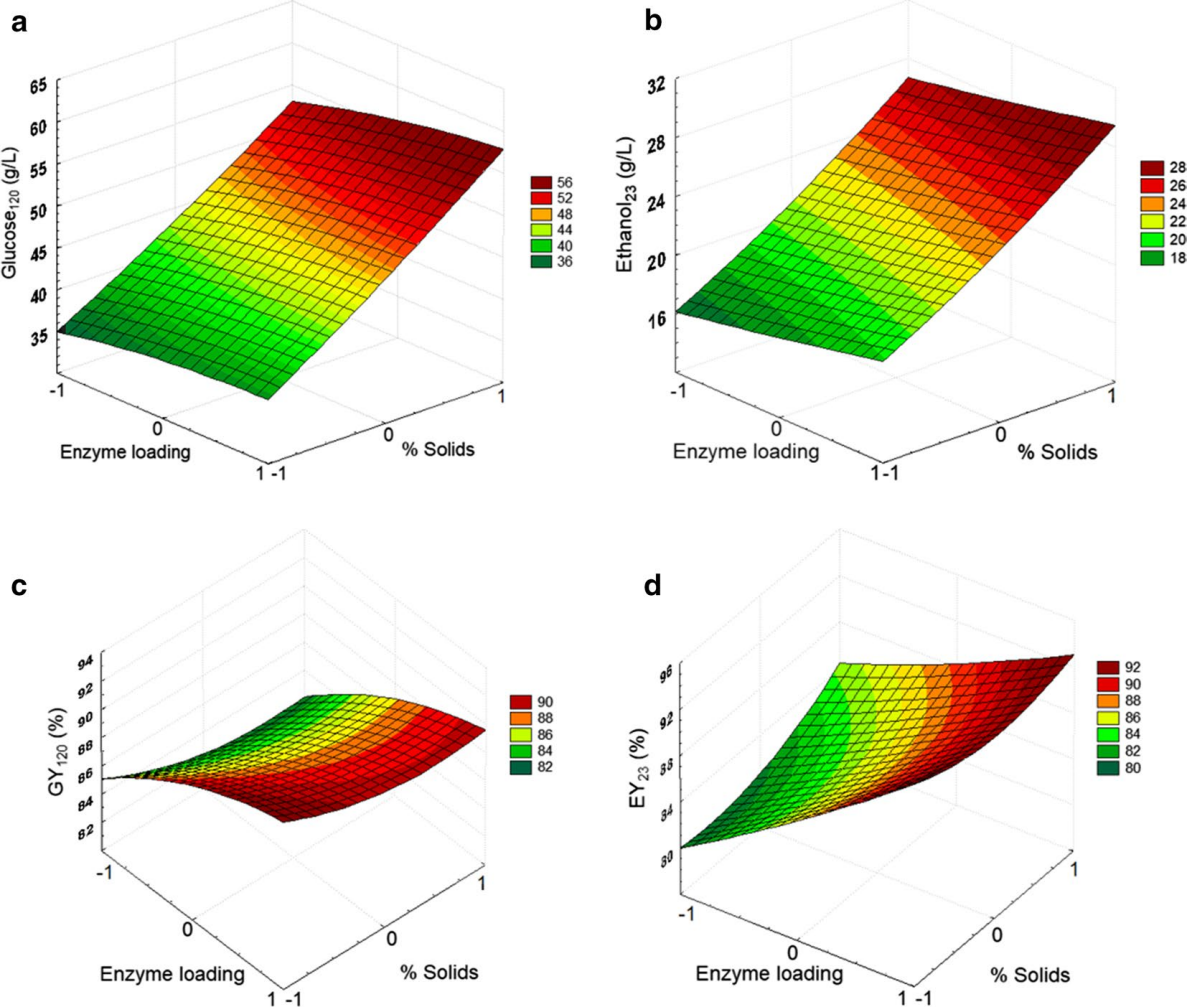
Response surfaces for Glu_120_ (**a**), Eth_23_ (**b**), GY_120_ (**c**), and EY_23_ (**d**) as a function of solid concentration (*X*_1_) and enzyme loading (*X*_2_)

From ANOVA analysis, it was verified that these models adequately represent the values of Glu_120_, GY_120_, Eth_23_ and EY_23_, with an estimated determination coefficient (*R*^2^) of 0.989, 0.824, 0.989 and 0.877, respectively. *F* value was higher than the tabular *F* (3.33) for all the models, indicating that they are statistically significant for a confidence level of 95%. Additionally, the non-significant values of lack of fit also suggest an adequate fitting of the different models (Table 4). For each model, the correspondent response surface was constructed to better visualize the influence of each variable on the different responses (Fig. 4).

**Table 4:** Regression indicators and analysis of variance (ANOVA) for the different models

Indicator	Glu_120_	Eth_23_	GY_120_	EY_23_
*p* value				
*X*_1_	4.79E−11	4.91E−11	0.04175	0.18523
*X*_2_	0.00017	1.60E−11	9.122E−5	1.090E−5
*X*_1_^2^	0.38003	0.13104	0.26593	0.21956
*X*_2_^2^	0.32261	0.78432	0.32293	0.73420
*X*_1_*X*_2_	0.19371	0.96022	0.82280	0.19477
*F* value (model)	180.660	184.940	9.36420	14.3010
Significance *F*	1.82E−9	1.62E−9	0.00156	0.00028
*F* value (lack of fit)	0.89019	2.00101	0.68907	1.93724
*R* ^2^	0.98905	0.98930	0.82401	0.87731
${R^2}_{\text{adj}}$	0.98358	0.98395	0.73601	0.81596

Considering first the concentration of solids (*X*_1_), as expected, a significant positive (linear) effect was observed on both glucose and ethanol concentrations (*p* value of 4.8 × 10^−11^ and 4.9 × 10^−11^, respectively), justified by an increased availability of cellulose and fermentable sugars, respectively. Furthermore, there were no evidences of critical limitations caused by the high amounts of solids, namely mass transference related or end-product inhibition. That could be also observed from the model of glucose production yield (Fig. 4c), where no clear negative effect is visible; indeed, the glucose yield varies around values of 84–91%, a clear trend associated to solid content being unnoticeable. Very high solid concentrations are reported to have a significant negative impact on glucose yield, an effect that is not observed in this case since the range of solid concentration used was selected in exploratory assays. Also, it is worth noting that the hydrolysis was conducted for 120 h, which is the time required for satisfactory yields to be reached under the highest solid loadings, attenuating therefore time-dependent limitations. In a similar way, also the utilization of this specific range of enzyme loadings may have contributed to attenuate limitations resulting from increased solid loadings such as non-productive binding of enzymes to the solid. These results suggest that further intensification may still be achievable at industrial scale, using better mixing conditions than the ones available at lab scale in this study.

Finally, it still should be highlighted that, as the solid has a negative cost on this case, more important than the production yield is the productivity, equally critical for lowering operational costs. We can therefore consider 22% solids as the most adequate option under the lab scale setup available, as it leads to satisfactory glucose yield, enabling the maximum glucose concentration.

Reporting now to the influence of enzyme loading, although a slight increase is visible for all response variables it is not expressive. Additionally, it seems to impact similarly in the entire range of solid concentration, while a superior effect would be expected for the highest consistency where possible enzyme limitations would be more likely. Thus, it seems that for this range of solid and enzyme loadings there is indeed no significant limitation of enzyme availability. From a previous work by our group, it was verified that this specific cellulase cocktail is particularly efficient on the hydrolysis of nRPS [8].

Maximum values of glucose concentration were achieved for the highest level of enzyme dosage, as expected (Table 2). However, when enzyme dosage was increased in 50% (from 20 to 30 FPU/g_cellulose_) for the highest solid concentration, glucose concentration only increased approximately 12% (from 52.4 to 58.9 g/L). Considering the high cost of enzymes and negative cost of the substrate, a lower enzyme dosage may be a sensible choice in this scenario.

### nRPS hydrolysis with cellulase recycling under high solid loadings

Taking in account the results from CCI design, we envisage the nRPS conversion to high ethanol concentrations while enabling cellulase recycling. Hence, a system of multiples rounds of hydrolysis was implemented with Celluclast, applying the pre-determined conditions of solid and enzyme loadings.

**Fig. 5:**
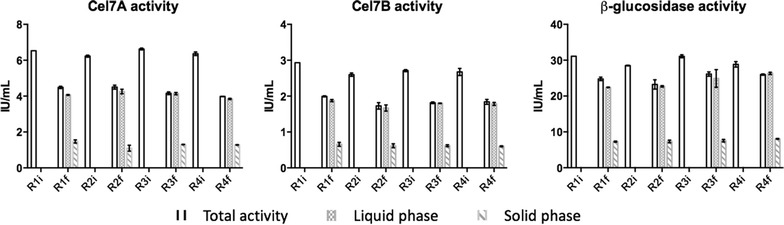
Variation of Cel7A, Cel7B and β-glucosidase activities over four rounds of nRPS hydrolysis (120 h hydrolysis [40 °C] → 24 h SSF [35 °C]) with cellulase recycling. 20 FPU/g_cellulose_ were initially employed with a posterior supplementation of 50% fresh enzymes on each recycling stage (Rxi and Rxf refers to the initial and final activity of round *x*, respectively)

From the analysis of Fig. 5, we may observe that the initial levels of the three cellulases analyzed (Cel7A, Cel7B and β-glucosidase) were similar over the four rounds of hydrolysis and fermentation, an outcome that could be achieved using a 50% supplementation with fresh enzymes in each round. As a matter of fact, for each round there is a considerable decrease on the activity levels, an average reduction of 33.4, 32.4 and 16.1% being observed for Cel7A, Cel7B and β-glucosidase, respectively. A lower reduction observed for β-glucosidase may be attributed to its well known lack of cellulose-binding domain. Also, the fact that β-glucosidase levels may have been used in excess, enables an inferior relative variation. Referring to a previous work, the levels of activity variations for this case were considerably higher comparing to average decreases of 14.3, 17.6 and 7.0% obtained for Cel7A, Cel7B and β-glucosidase, respectively [8]. Considering that there was no thermal deactivation, it may be possible that the higher concentrations of ethanol achieved on this case may have caused some loss of enzyme activity [37] since the intensification strategy followed in the present study allowed a 3.8-fold increase in ethanol concentration.

Referring to the enzyme distribution at the end of each cycle, the results demonstrate that a considerable fraction of activity remained solid-bound: an average of 30.4, 32.6 and 30.3% for Cel7A, Cel7B and β-glucosidase, respectively. This result highlights the need to recover both fractions in spite of increasing process complexity.

From the steady levels of initial activity for the different cellulases along the different cycles, one could expect the applied strategy of cellulase recycling to achieve equal levels of solid conversion along the process. Nevertheless, it was verified that hydrolysis efficiency had an average decrease of 12.5% in the rounds with recycled enzyme comparatively to the initial one (Table 5). A major part of this reduction may possibly arise from a different sterilization process used. While the first nRPS batch was sterilized after being suspended in the liquid (approx. 22% solids), the following ones were processed at high consistency (approx. 95% solids), which leads to a decrease on solid conversion by around 14%. This was required to enable a higher volume of concentrate after ultrafiltration since high final enzyme concentrations have shown before to cause higher losses during this process. On an industrial scale, however, the utilization of different sterilization processes or UF devices with lower limitations may enable to overcome in some degree this reduction. In addition, this decrease may equally be attributed to the fact that on rounds 2, 3 and 4, 50% of the enzymes have already undertaken at least one cycle of hydrolysis and fermentation, which can cause to some extent a reduction on their efficiency.

**Table 5:** Multiple rounds of nRPS hydrolysis with cellulase recycling (20 FPU/g cellulose; 50% fresh enzymes)

Round	Glucose_120_ (g/L)^a^	Ethanol_23_ (g/L)^b^	Glucan conversion (%)
1	50.14 ± 0.55	25.86 ± 0.67	80.68 ± 0.44
2 (recycling 1)	41.86 ± 1.06	20.94 ± 0.85	70.55 ± 1.34
3 (recycling 2)	42.31 ± 0.76	21.39 ± 0.08	70.26 ± 0.13
4 (recycling 3)	40.74 ± 0.36	20.28 ± 0.15	70.18 ± 0.35

Hydrolysis was conducted for 120 h (40 °C) followed 24 h of fermentation (35 °C)

^a^Glucose produced at 120 h of hydrolysis

^b^Ethanol produced at 23 h of fermentation

In spite of this decrease on hydrolysis efficiency, we should highlight that it was still possible to reach important improvements in both glucose and ethanol production comparatively to the existing literature. Using a similar substrate (although with slightly superior cellulose content), the maximum ethanol concentration obtained by Marques et al. [29] was 19.6 g/L. Also, Marques et al. [38] were able to achieve nearly 80 g/L of glucose; nevertheless, this was obtained through a fed-batch strategy with multiple pulses of substrate addition and not a single addition as for the current work. Comparing specifically to a previous work also applying cellulases recycling on RPS conversion [8], it was verified an increase of 3.4- and 3.8-fold on glucose and ethanol productions, respectively. Even employing a set of much more challenging conditions to the process, namely a higher temperature of hydrolysis and fermentation and a considerable increase on solid loading, it was still possible to successfully implement the recycling of cellulases enabling an approximate enzyme saving of 50%, to nearly 10 FPU/g_cellulose_. It should be referred that when hydrolysis was conducted in the same conditions as for the cycles with recycled enzyme but using instead only 10 FPU/g_cellulose_ (simulating the estimated enzyme saving) glucose production decreased approximately 35% (from 41.6 to 27.0 g/L).

## Conclusions

This work provides critical insights from the perspective of a future industrial implementation of enzyme recycling in the specific case of bioethanol production from RPS. It demonstrates that this material can be efficiently converted by different commercial cocktails currently available even under intensified conditions. Also, it elucidates the important role of enzyme cocktail selection on determining the final distribution of enzymatic activity between phases and its overall recovery after the process, a critical factor on the establishment of a simple recycling strategy. In this scope, Celluclast showed a more favorable scenario comparatively to other cocktails, enabling as well a slight advantage on the hydrolysis efficiency.

Even employing intensified operational conditions, cellulase recycling was successfully implemented on RPS conversion with the addition of only 50% of enzymes on each recycling stage, suggesting that process intensification may be combined with enzyme recycling.

## Footnotes

1 ACCELLERASE is a registered trademark of Danisco US Inc. or its affiliated companies.

2 In providing samples to the authors, DuPont does not endorse the results, conclusions, and/or views expressed herein, and makes no representation and/or warranty concerning the samples, including, but not limited to, the availability of samples for research or commercial purposes, merchantability, fitness for a particular purpose and/or noninfringement of intellectual property rights.
